# Early non-neutralizing, afucosylated antibody responses are associated with COVID-19 severity

**DOI:** 10.1126/scitranslmed.abm7853

**Published:** 2022-01-18

**Authors:** Saborni Chakraborty, Joseph C. Gonzalez, Benjamin L. Sievers, Vamsee Mallajosyula, Srijoni Chakraborty, Megha Dubey, Usama Ashraf, Bowie Yik-Ling Cheng, Nimish Kathale, Kim Quyen Thi Tran, Courtney Scallan, Aanika Sinnott, Arianna Cassidy, Steven T. Chen, Terri Gelbart, Fei Gao, Yarden Golan, Xuhuai Ji, Seunghee Kim-Schulze, Mary Prahl, Stephanie L. Gaw, Sacha Gnjatic, Thomas U. Marron, Miriam Merad, Prabhu S. Arunachalam, Scott D. Boyd, Mark M. Davis, Marisa Holubar, Chaitan Khosla, Holden T. Maecker, Yvonne Maldonado, Elizabeth D. Mellins, Kari C. Nadeau, Bali Pulendran, Upinder Singh, Aruna Subramanian, Paul J. Utz, Robert Sherwood, Sheng Zhang, Prasanna Jagannathan, Gene S. Tan, Taia T. Wang

**Affiliations:** ^1^ Department of Medicine, Division of Infectious Diseases, Stanford University, Stanford, CA, 94304, USA.; ^2^ Program in Immunology, Stanford University School of Medicine, Stanford, CA, 94305, USA.; ^3^ J. Craig Venter Institute, La Jolla, CA, 92037, USA.; ^4^ Institute for Immunity, Transplantation, and Infection, Stanford University School of Medicine, Stanford, CA, 94305, USA.; ^5^ Department of Computer and Software Engineering, San Jose State University, San Jose, CA, 95192, USA.; ^6^ Department of Microbiology and Immunology, Stanford University School of Medicine, Stanford, CA, 94305, USA.; ^7^ Division of Maternal-Fetal Medicine, Department of Obstetrics, Gynecology, and Reproductive Sciences, University of California San Francisco, San Francisco, CA, 94143, USA.; ^8^ The Precision Immunology Institute, Icahn School of Medicine at Mount Sinai, New York, NY, 10029-5674, USA; ^9^ The Tisch Cancer Institute, Icahn School of Medicine at Mount Sinai, New York, NY, 10029-5674, USA; ^10^ Department of Oncological Sciences, Icahn School of Medicine at Mount Sinai, New York, NY, 10029-5674, USA; ^11^ Department of Bioengineering and Therapeutic Sciences, and Institute for Human Genetics, University of California San Francisco, San Francisco, CA, 94143, USA.; ^12^ Division of Pediatric Infectious Diseases, Department of Pediatrics, University of California, San Francisco, CA, 94143, USA.; ^13^ Human Immune Monitoring Center, Precision Immunology Institute, Department of Oncological Sciences, Icahn School of Medicine at Mount Sinai, New York, NY, 10029-5674, USA.; ^14^ Departments of Pathology and of Microbiology and Immunology, Stanford University School of Medicine, Stanford, CA, 94305, USA.; ^15^ Howard Hughes Medical Institute, Stanford University School of Medicine, Stanford, CA 94305, USA.; ^16^ Departments of Chemistry and Chemical Engineering, Stanford University, Stanford, CA, 94305, USA.; ^17^ Department of Pediatrics, Stanford University School of Medicine, Stanford, CA, 94305, USA.; ^18^ Sean N. Parker Center for Allergy and Asthma Research, Stanford, CA, 94304, USA.; ^19^ Department of Medicine, Division of Immunology and Rheumatology, Stanford University School of Medicine, Stanford, CA, 94304, USA; ^20^ Proteomics and Metabolomics Facility, Institute of Biotechnology, Cornell University, Ithaca, NY, 14853, USA.; ^21^ Division of Infectious Diseases, Department of Medicine, University of California San Diego, La Jolla, CA, 92093, USA.; ^22^ Chan Zuckerberg Biohub, San Francisco, CA, 94158, USA.

## Abstract

A damaging inflammatory response is implicated in the pathogenesis of severe coronavirus disease 2019 (COVID-19), but mechanisms contributing to this response are unclear. In two prospective cohorts, early non-neutralizing, afucosylated IgG antibodies specific to severe acute respiratory syndrome coronavirus 2 (SARS-CoV-2) were associated with progression from mild to more severe COVID-19. In contrast to the antibody structures that were associated with disease progression, antibodies that were elicited by mRNA SARS-CoV-2 vaccines were instead highly fucosylated and enriched in sialylation, both modifications that reduce the inflammatory potential of IgG. To study the biology afucosylated IgG immune complexes, we developed an in vivo model that revealed that human IgG-Fc gamma receptor (FcγR) interactions could regulate inflammation in the lung. Afucosylated IgG immune complexes isolated from COVID-19 patients induced inflammatory cytokine production and robust infiltration of the lung by immune cells. By contrast, vaccine-elicited IgG did not promote an inflammatory lung response. Together, these results show that IgG-FcγR interactions are able to regulate inflammation in the lung and may define distinct lung activities associated with the IgG that are associated with severe COVID-19 and protection against infection with SARS-CoV-2.

## Teaser

Divergent early antibody structures are associated with severe COVID-19 and are functionally distinct from mRNA vaccine-elicited antibodies in vivo.

## INTRODUCTION

The minority of people who develop severe coronavirus disease 2019 (COVID-19) during severe acute respiratory syndrome coronavirus 2 (SARS-CoV-2) infection mount an inflammatory response that is implicated in disease pathogenesis (*1*–*3*). The extreme inflammatory phenotype in the lungs of patients with severe COVID-19 is clear from autopsy studies, but mechanisms contributing to this response are not well understood (*4*–*7*). IgG antibodies mediate cellular functions that are central in directing the course of disease during many viral infections. Aside from neutralizing activity, IgG antibodies that bind to virus particles or viral antigens can form immune complexes (ICs) that may have an impact on disease pathogenesis, especially with regard to inflammation. This is observed in some autoimmune and infectious diseases where persistent ICs drive a hyperinflammatory response that damages host tissues (*8*). A clear mechanism underlying modulation of inflammation by antibodies is through IgG interactions with activating and inhibitory Fc gamma receptors (FcγRs) on myeloid cells, which are central regulators of the inflammatory response. We and others have previously found that patients with severe COVID-19 produce a high abundance of afucosylated IgG antibodies that trigger inflammatory responses in primary monocytes (*9*–*11*). This response was dependent on Fc afucosylation, a modification that enhances affinity of monomeric IgG for the activating FcγR, CD16a, by approximately 10-fold (*12*, *13*).

Because IgG ICs can promote disease sequelae in some infections, the link between severe COVID-19 and afucosylated IgG suggests that this antibody type may have a role in the inflammatory pathogenesis of severe disease. To explore this, we first studied whether afucosylated antibody production was a consequence of, or an antecedent to, the development of more severe COVID-19. In two independent cohorts assessed during an initial period of mild symptoms, we found that the absence of early neutralizing antibodies, together with an increased abundance of afucosylated IgG, was associated with rapid progression to more severe disease. Elevated frequencies of monocytes expressing the receptor for afucosylated IgG, CD16a, were also associated with more severe outcomes. To study the effect of afucosylated antibody signaling in the lungs, we developed a model system in which human ICs of defined composition are intratracheally administered to mice that express human FcγRs (*14*). Molecular and cellular changes that were triggered in the lung by distinct antibody signaling pathways were then assessed by characterization of bronchoalveolar lavage (BAL) fluid collected after IC administration. This model provided a physiologically relevant system to study antibody effector responses in the lung. We observed that afucosylated ICs triggered robust immune cell activation, infiltration into the lungs, and proinflammatory cytokine production that was CD16a-dependent. In contrast to infection, SARS-CoV-2 mRNA vaccination elicited IgG antibodies that were both highly fucosylated and sialylated. Immune complexes formed from mRNA vaccine-elicited IgG did not trigger the cellular infiltration or the cytokine and chemokine production that were associated with afucosylated IgG in vivo. Overall, these findings demonstrate that early production of non-neutralizing, afucosylated IgG1 was associated with COVID-19 symptom progression; these antibodies were structurally and functionally distinct from IgG1 elicited by mRNA SARS-CoV-2 vaccination.

## RESULTS

### Study cohorts

To study the early antibody features that correlated with different disease outcomes in COVID-19, we characterized IgG from two longitudinal cohorts of COVID-19 outpatients from Stanford Hospital Center (n=109 Cohort 1 at enrollment; n=69 Cohort 2). Although these samples were collected from interventional clinical studies, we evaluated data only from the placebo arm of both studies; thus, our findings are not impacted by the experimental treatments trialed in either study. Participants in both studies were enrolled early in infection, within three days of a positive SARS-CoV-2 polymerase chain reaction (PCR) test. All participants presented with mild COVID-19 and had mild symptoms at the time of enrollment, as determined by a physician’s assessment (*15*). Although uncomplicated resolution of mild disease occurred in the majority of participants, a subset of patients in each cohort (n=8 in Cohort 1; n=7 in Cohort 2) developed worsening symptoms in the hours or days following enrollment. These individuals were evaluated in the emergency department and some required hospitalization; one individual succumbed to disease. We term these patients with distinct disease trajectories as “progressors” (tables S1, S2, S3) or “non-progressors”. Progressors and non-progressors from Cohort 1 did not differ by the parameters of age, weight, or sex. Progressors from Cohort 2 also did not differ based on weight or sex but were older compared to non-progressors (table S1).

### Low early neutralizing IgG responses were observed in progressors.

**Fig. 1.**
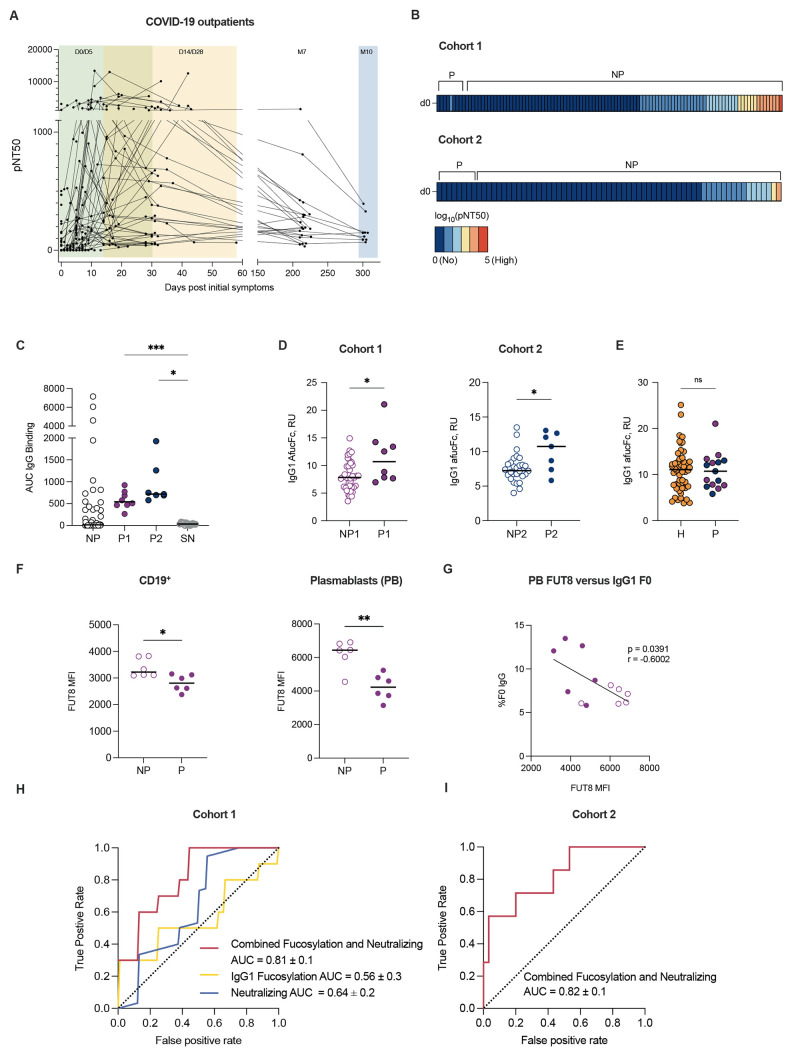
**Low early neutralizing titers and elevated Fc afucosylation are associated with COVID-19 progression**. (**A**) The kinetics of neutralizing antibody responses were measured over time in Cohort 1. Half-maximal SARS-CoV-2 pseudovirus neutralizing titers (pNT50) are shown at each study time point, graphed based on days of symptoms for each participant. Samples were collected at study day 0 (D0 enrollment; n=101), 5 (D5; n=50), 14 (D14; n=33), 28 (D28; n=43), month 7 (M7; n=24) and month 10 (M10; n=9). (**B**) Heatmaps of pNT50 data are shown for progressors (P) (Cohort 1 n=8, Cohort 2 n=7) and non-progressors (NP) at enrollment timepoint (D0). The scale ranges from dark blue (no neutralization) to red (high neutralization). (**C**) SARS-CoV-2 spike-binding IgG (AUC) are shown for Cohort 1 progressors (P1, solid purple), Cohort 2 progressors (P2, solid blue), a random subset of non-progressors, and historic seronegative (SN) serum samples. (**D**) IgG1 Fc afucosylation abundance was measured in samples from progressors and non-progressors at enrollment timepoint (D0) of Cohort 1 (purple; progressors=P1, non-progressors=NP1) and in samples from Cohort 2 (blue; progressors=P2, non-progressors=NP2). RU, relative units. (**E**) IgG1 Fc afucosylation abundance was measured in patients who were hospitalized with COVID-19 (H, orange; n=52) and combined outpatient progressors (P, Cohort 1 progressors: purple), Cohort 2 progressors: blue) (n=15). (**F**) α-1,6-Fucosyltransferase 8 (FUT8) median fluorescence intensity (MFI) was measured in total CD19^+^ B cells and in plasmablasts (PB) from progressors (n=6) relative to sex-matched non-progressors (n=6). (**G**) The correlation for plasmablast expression of FUT8 and the abundance of IgG1 afucosylation is shown for matched samples. Solid and open circles represent data points from progressors and non-progressors, respectively. (**H**) Mean receiver operating characteristic (ROC) response and the area under the curve (AUC) with its standard deviation were obtained with a support vector machine classifier (SVM) using neutralization titers and IgG1 afucosylation. (**I**) ROC response and the AUC with standard deviation were obtained by testing the model on an independent Cohort 2. Median values are depicted in (C to F) with a solid black line. P values in (C) were calculated using Brown-Forsythe and Welch ANOVA test with Dunnett T3 correction, P values in (D and E) were calculated using Wilcoxon rank-sum test, and P values in (F) were calculated using unpaired Student’s test with Welch’s correction. *P < 0.05; **P < 0.01; ***P < 0.001; ns, not significant. Pearson’s correlation coefficient (r = -0.6002, p = 0.0391) was computed in (G).

The availability of samples from the date of enrollment in both studies (here termed “day 0”), when all participants had mild disease, enabled our analysis of early antibody responses that correlated with distinct disease trajectories. We first defined the evolution of the neutralizing antibody response following SARS-CoV-2 infections using a pseudotyped vesicular stomatitis virus neutralization assay. The fifty percent pseudoviral neutralizing antibody titers (pNT50) were calculated for day 0, day 5, day 28, month 7, and month 10 for all participants in the placebo arm of Cohort 1 from whom samples were available. Samples from study participants who received a SARS-CoV-2 vaccine within the study period were not evaluated. In most individuals, abundance of neutralizing antibodies showed an increase over time, peaking by day 28. Once initiated, the antibody response was durable and persisted in most people until 7 months post-enrollment, after which there was a general decrease in neutralization by month 10 (Fig. 1A, fig. S1A). This analysis of Cohort 1 revealed that, although there was considerable heterogeneity in early neutralizing responses, those participants who would progress to more severe disease had uniformly very low or no detectible neutralizing antibodies at the study enrollment time point (Fig. 1B). Cohort 2 showed somewhat less heterogeneity in early neutralizing responses, but as with Cohort 1, neutralizing antibodies were not detected on day 0 in any of the progressors (Fig. 1B). These data were broadly consistent with studies showing a correlation between early neutralizing antibody responses and outcomes in COVID-19 (*16*–*19*).

We initially reasoned that the absence of early neutralizing antibodies in progressors might have been due to earlier sampling of participants who were on a more severe disease trajectory. To evaluate this, we compared the number of symptomatic days prior to study enrollment in progressors and non-progressors. This revealed that there were no significant differences in the mean or median duration of symptoms prior to enrollment (*P* > 0.05, table S1). Thus, the kinetics of sampling did not explain this observation. Despite the absence of early neutralizing responses, SARS-CoV-2 spike-reactive IgG was present in all progressors (Fig. 1C). Although early neutralizing responses were not detected, progressors from whom longitudinal samples were available generally mounted neutralizing antibody responses by the later study timepoints (fig. S1B).

### Elevated early production of afucosylated IgG was observed in progressors.

We next asked whether there were qualitative differences in the Fc structures of the IgG in progressors and non-progressors. As we had previously observed elevated anti-SARS-CoV-2 Fc afucosylation in hospitalized patients compared to outpatients (*9*), we sought to clarify whether these antibodies were produced in response to severe disease or whether they might precede the development of severe symptoms. To study this, we evaluated Fc glycosylation on antibodies present at study enrollment when all individuals had mild symptoms. Indeed, at study enrollment the progressors in both cohorts were already distinguished by an elevated abundance of afucosylated IgG1, comparable to the elevated abundance observed in a cohort of hospitalized COVID-19 patients in the Mount Sinai Health System (Fig. 1D, E, table S1) (*20*). We observed no correlations between demographic features and IgG afucosylation in either outpatient cohort (fig. S1C). The abundance of afucosylated IgG1 in COVID-19 outpatients was not different across timepoints that were separated by approximately 200 days (fig. S1D). These data show that production of afucosylated IgG preceded the onset of severe symptoms and afucosylated antibodies were maintained over time.

We next sought to investigate the basis of differences in antibody fucosylation. We hypothesized that differences in expression of the relevant glycosyltransferase, α-1,6-fucosyltransferase (FUT8), by antibody-secreting cells, might play a role. To investigate this, we assessed FUT8 protein abundance in peripheral blood mononuclear cells (PBMCs) from progressors and non-progressors (fig. S2A). At the time of this experiment, only PBMCs from Cohort 2 were available from the enrollment timepoint. Because we have previously observed a sex-based difference in antibody afucosylation (*9*), an equivalent number of sex-matched non-progressors were selected for this analysis. As previously mentioned, no other correlations between demographic features and IgG afucosylation were observed in either cohort (fig. S1C). Consistent with the elevated production of afucosylated IgG by progressors, CD19^+^ B cells and plasmablasts from progressors expressed less FUT8 than cells from non-progressors upon enrollment (Fig. 1F). FUT8 expression within total PBMCs was comparable between groups, as was the distribution of B cell subsets, suggesting that FUT8 expression was regulated at the effector cell level (fig. S2A to C). Of note, plasmablast expression of FUT8 correlated with IgG1 Fc afucosylation, supporting the hypothesis that IgG afucosylation is regulated, at least in part, by the expression of FUT8 (Fig. 1G).

### Early non-neutralizing, afucosylated anti-spike IgG were associated with worsening symptoms in COVID-19 outpatients.

To determine whether the combination of low or no neutralizing antibodies and elevated IgG Fc afucosylation was associated with worsening disease trajectory in patients with mild COVID-19, we next trained and evaluated a support vector machine (SVM) classifier by using day 0 neutralization titers and afucosylated IgG frequency as input features from Cohort 1. Individually, both early neutralization titers and Fc afucosylation had low to modest predictive power to separate progressors and non-progressors, whereas combining the two features could separate progressors from non-progressors with higher predictive accuracy (Fig. 1H). Subsequently, the Cohort 1 data was used as the training set and the performance of the model was evaluated on an independent test dataset (Cohort 2). As shown, the combined features could discriminate divergent disease outcomes with area under the receiver operating characteristic (ROC) curve (AUC) of 0.81 (Fig. 1I). Thus, early production of reactive, afucosylated antibodies and poor serum neutralizing activity may be able to predict progression from mild COVID-19 to more severe outcomes.

### The receptor for afucosylated IgG1, CD16a, is enriched in the myeloid compartment of progressors

In addition to afucosylated antibody production, a hallmark of patients with severe COVID-19 is inflammatory myeloid cell infiltration into the lung and excessive inflammatory cytokine production (*2*, *21*, *22*). These cells express the low affinity FcγRs CD32a (activating), CD32b (inhibitory) and, on some subsets, CD16a (activating). These low affinity FcγRs are engaged through avidity-based interactions when ICs are formed during infection. Depending on the magnitude of activating or inhibitory signal received upon engagement, an effector cell will respond with a proportional degree of inflammatory activity (*9*). Considering that severe COVID-19 is often characterized by an aberrant effector cell activation state (*1*, *23*–*25*), we next sought to define the expression of activating and inhibitory FcγRs on peripheral monocytes from progressors and non-progressors that might counterbalance or compound an enrichment of afucosylated IgG.

**Fig. 2.**
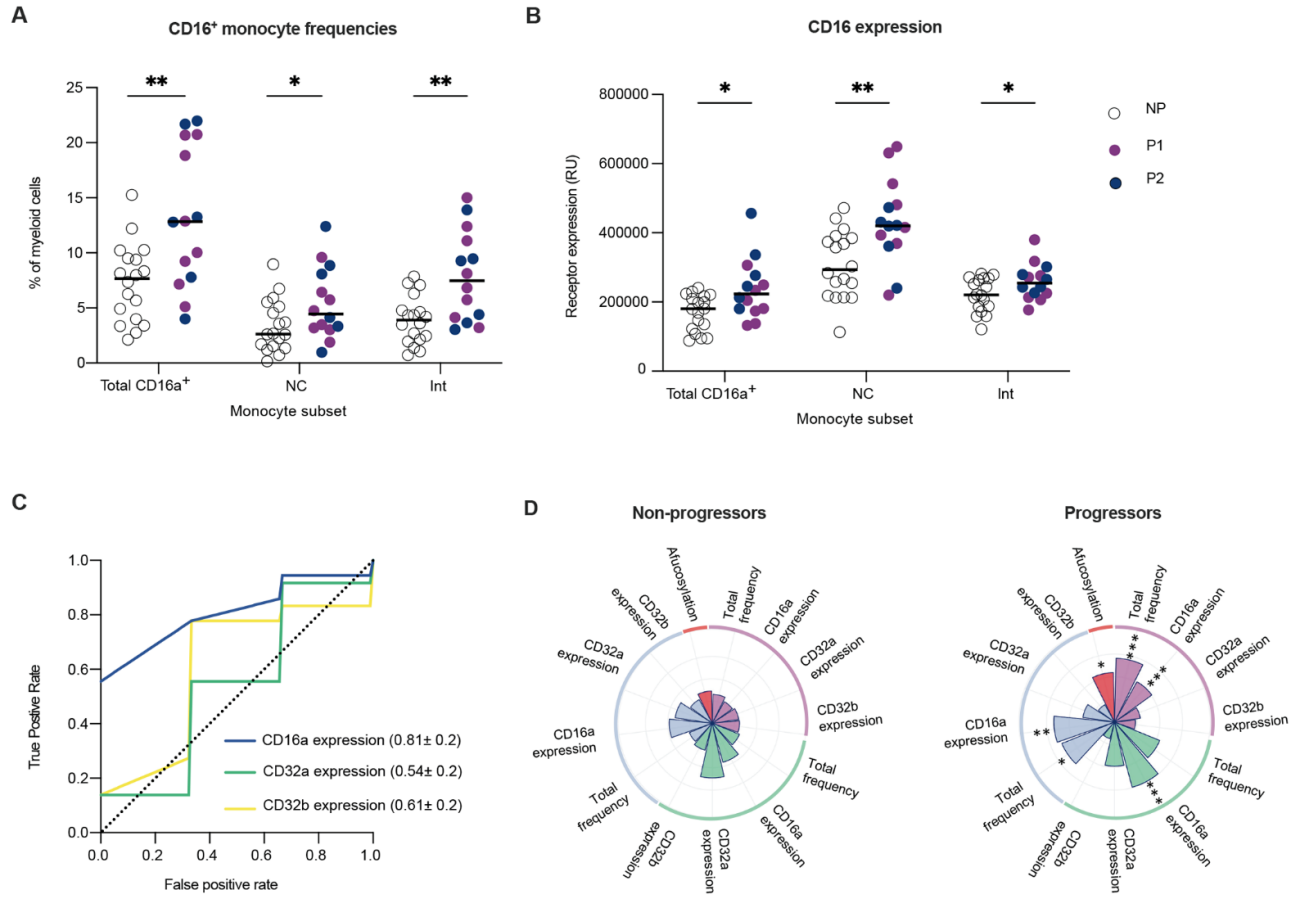
**CD16a signaling potential is elevated in the myeloid compartment of progressors**. Enrollment time point PBMCs were characterized in progressors (n=14) and a randomly selected subset of non-progressors (n=18). Solid purple and blue circles represent data points from progressors within Cohort 1 and Cohort 2, respectively whereas open circles represent data points from non-progressors. The median values have been depicted with a black line. (**A**) Total CD16a^+^ monocyte, CD16a^+^ CD14^-^ non-classical monocyte (NC), and CD16a^+^ CD14^+^ intermediate monocyte (Int) frequencies are shown as percentages of total CD11c^+^ HLA-DR^+^ CD3^-^ CD19^-^ CD56^-^ myeloid cells. (**B**) CD16a expression was measured on total CD16a^+^, non-classical, and intermediate monocyte populations. Receptor expression is measured in relative units (RU) (**C**) Mean ROC response and the AUC with its standard deviation were obtained using random forest classifier with 6-fold cross validation in two outpatient cohorts using FcγR expression on myeloid cells. (**D**) Radar plots summarizing the various features of IgG1-CD16a signaling axis in progressors and non-progressors are shown. Significant differences between the two groups are indicated with asterisks in the radar plot for progressors. P values in (A and B) were calculated using unpaired *t* tests with Welch’s correction. *P < 0.05, **P < 0.01, ***P < 0.001.

To study this, available PBMC samples collected at study enrollment were assessed for the frequency of CD16a-expressing monocyte subsets, as well as their expression of all low affinity FcγRs (fig. S3A). Notably, we found that progressors had increased frequencies of total CD16a^+^ monocytes, CD16a^+^ CD14^-^ non-classical monocytes, and CD16a^+^ CD14^+^ intermediate monocytes within the peripheral CD11c^+^ HLA-DR^+^ myeloid cell compartment compared to non-progressors upon study enrollment (Fig. 2A) (*26*–*28*). Further, quantitative expression analysis of CD16a within these immune cell subsets revealed higher expression of CD16a on cells from progressors, whereas other low affinity FcγRs (CD32a/b) were not differentially expressed (Fig. 2B, fig. S3B). Taken together, early CD16a expression within the peripheral myeloid cell compartment was associated with the development of more severe symptoms in COVID-19 outpatients (Fig. 2C and D).

### mRNA vaccination elicits the production of neutralizing IgG with glycoforms that are distinct from those elicited by infection.

**Fig. 3.**
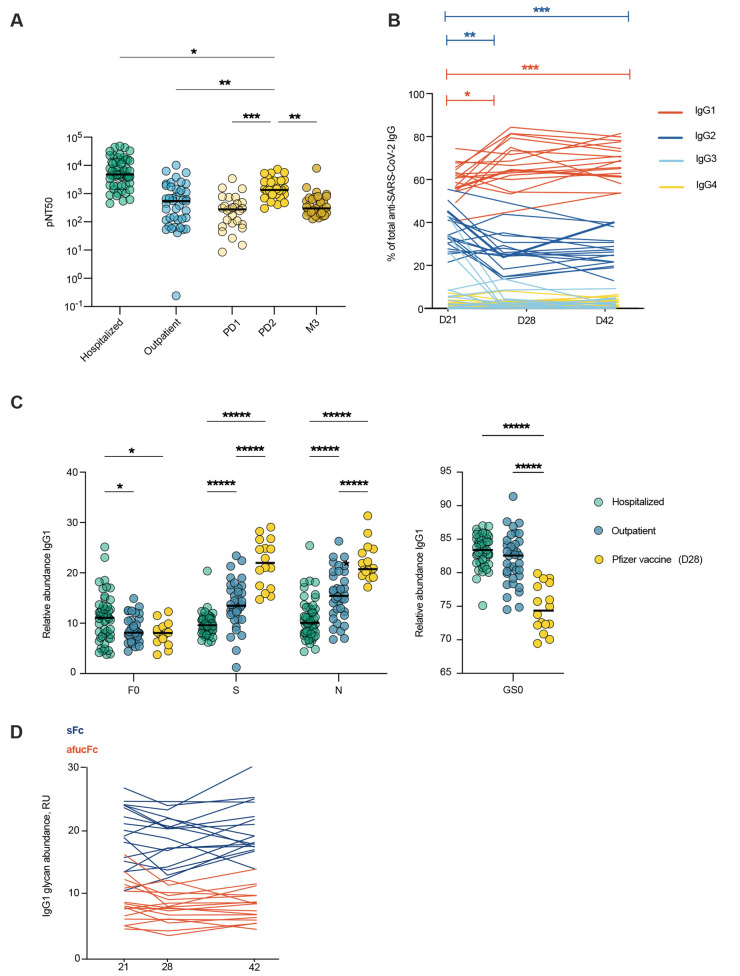
**mRNA vaccination elicits high neutralizing antibody titers with Fc glycoforms distinct from infection-induced IgG phenotypes**. (**A**) The half-maximal SARS-CoV-2 pseudovirus neutralizing titers (pNT50) in healthy adults following mRNA vaccination (yellow n=29) or in COVID-19 outpatients on study day 28 (blue n=42) are shown. PD1: post-dose 1, PD2: post-dose 2, M3: month 3. (**B**) Longitudinal analysis of IgG subclasses is shown for day 21, 28, or 42 post-primary vaccination (n=17). (**C**) SARS-CoV2 IgG1 Fc posttranslational modifications were analyzed in samples from patients hospitalized with COVID-19 (n=52), COVID-19 outpatients (day 28 n=36) and in participants who received the Pfizer BNT162b2 SARS-CoV-2 mRNA vaccine (day 28 post primary vaccination, n=16). F0: afucosylation, S: sialylation, N: bisection, GS0: galactosylation. (**D**) Longitudinal analysis of anti-SARS-CoV-2 IgG1 Fc afucosylation (afucFc, red line) and sialylation (sFc, blue line) is shown on day 21, 28 or 42 post-primary vaccination. The median values in (A and C) are depicted with a black line. P values in (A) were calculated using Kruskal Wallis test with Dunn’s correction, in (B) using mixed effect analysis with Geisser-Greenhouse and Tukey’s corrections, and in (C) using a two-way ANOVA and one-way ANOVA with Tukey’s correction. *P < 0.05; **P < 0.01; ***P < 0.001; ****P < 0.0001.

We next sought to compare the quality of antibodies produced after SARS-CoV-2 mRNA vaccination and infection. To do so, we studied the antibodies elicited after 1 and 2 doses of the Pfizer BNT162b2 SARS-CoV-2 mRNA vaccine in a group of healthy SARS-CoV-2-naïve adults (Stanford adult vaccine cohort, n=29) (table S4). Neutralizing titers increased between the post-primary vaccination timepoint (21 days post-dose 1 (PD1)) and the post-does 2 timepoint (21 days post-dose 2 (PD2)) (Fig. 3A). In all, two doses of mRNA SARS-CoV-2 vaccine elicited robust neutralizing antibody responses that were elevated over peak outpatient neutralizing titers (day 28 shown) (Fig. 3A). Over time after vaccination, the distribution of anti-spike IgG subclasses shifted to a more dominant proportion of IgG1 antibodies (Fig. 3B).

We next characterized Fc glycoforms of anti-spike IgG to determine whether infection- and mRNA vaccine-elicited IgG were distinct in this respect (*10*). For this analysis, IgG from day 28 of the outpatient COVID-19 study (from non-progressors) were compared to samples drawn from vaccine recipients on day 28 post-primary vaccination (7 days PD2). Additionally, we compared these groups to IgG samples from a cohort of individuals hospitalized with COVID-19 (Mount Sinai). Abundance of IgG1 Fc afucosylation were similar between the outpatient and vaccine-elicited IgG (Fig. 3C) and both groups were reduced in afucosylation relative to hospitalized patients. Interestingly, vaccination-induced IgG was enriched in Fc sialylation over IgG from outpatients and individuals hospitalized with COVID-19, suggesting differential regulation of Fc sialylation by mRNA vaccination and infection, though we cannot exclude a contribution from demographic features that were not matched between cohorts. The relative homogeneity of Fc glycosylation in response to mRNA vaccination contrasted with the heterogeneity observed in infection, as well as with our previous observations after seasonal influenza virus vaccination, suggesting differences in the response that may be based on the context of antigen encounter, antigen experience, or different vaccine platforms (*29*). Vaccine-elicited Fc afucosylation and sialylation were relatively stable over time, similar to the stability observed after infection (Fig. 3D, fig. S1C). Thus, SARS-CoV-2 infection and mRNA vaccination both elicited high neutralizing titers, but with distinct and stable abundances of IgG1 Fc afucosylation and sialylation.

### Afucosylated immune complexes trigger inflammation in the lung in vivo.

To study the functional relevance of the differential glycosylation of mRNA- and infection-elicited IgG, we established an in vivo experimental model designed specifically to enable dissection of human antibody signaling outcomes in the lung in the absence of any additional effects imposed by infection. In this model, pre-formed human IgG ICs, simulating what would be formed during an infection, are delivered to lungs of mice that express human, instead of murine, FcγRs, with cell-specific distribution that recapitulates the human system (*14*). Polyclonal IgG pools were generated from purified serum IgG. Pools were from patients with elevated (pool 1, >20%) or normal abundance (pool 2, <10%) of afucosylated IgG or from serum isolated from mRNA-vaccinated adults (pool 3). Pools 1 and 2 did not differ in other glycan modifications, and all 3 pools exhibited comparable distribution of IgG subclasses, though pool 3 tended to have lower IgG1 and higher IgG2 content (fig. S4A and B). All IgG pools were standardized for binding to SARS-CoV-2 spike (fig. S4C). Mice were intratracheally administered ICs composed of the anti-SARS-CoV-2 IgG and trimeric SARS-CoV-2 spike protein. Four hours following IC administration, contents of BAL fluid were analyzed for immune cells and soluble factors. This system provided a context in which to specifically study how modulation of IgG Fc-FcγR interactions impacts the immune response in the lung.

**Fig. 4.**
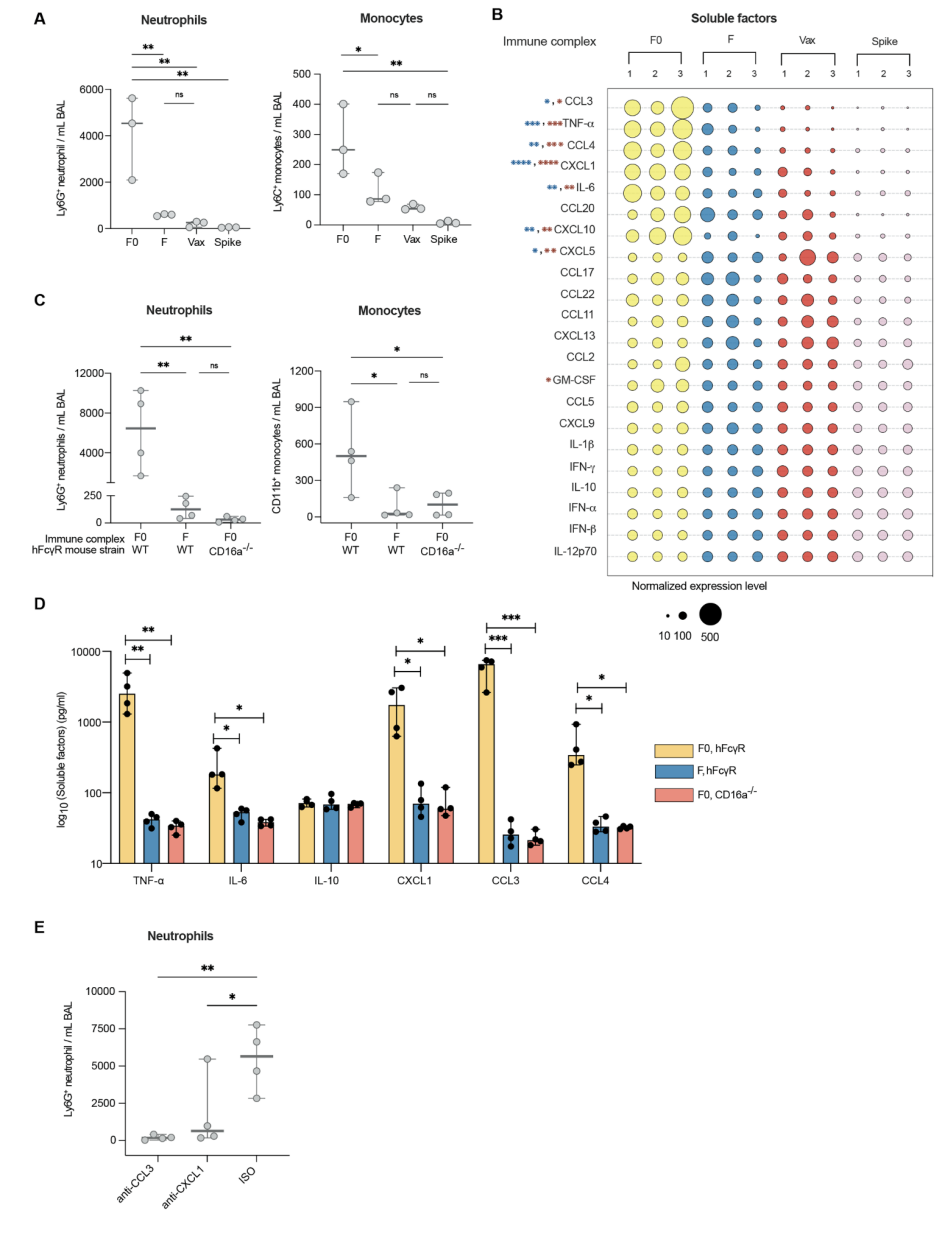
**Afucosylated IgG immune complexes promote immune cell infiltration and proinflammatory cytokine production in vivo**. (**A**) Immune cells were measured in the bronchoalveolar lavage (BAL) fluid of human Fcγ receptor mice (hFcγR) that were treated with either afucosylated (F0, pool 1), normally fucosylated (F, pool 2), or vaccine-induced (Vax, pool 3) immune complexes or with spike protein alone. Immune complexes and spike protein were administered by the intratracheal route. (**B**) Cytokine and chemokine concentrations in the BAL of the indicated groups of mice are shown. The size of the bubble represents normalized cytokine and chemokine concentrations. P values are indicated for each soluble factor (blue: F0 versus F, red: F0 versus Vax). (**C** and **D**) Immune cell subsets (C) as well as cytokines (TNF-α, IL-6, IL-10) and chemokines (CXCL1, CCL3, CCL4) (D) were quantified in the bronchoalveolar lavage (BAL) fluid of hFcγR or hFcγR mice with a specific deletion in CD16a (CD16a^−/−^) that received afucosylated (F0, pool 1) or normally fucosylated (F, pool 2) immune complexes by intratracheal administration. In (A) and (C) neutrophils were defined as Ly6G^+^ CD11b^+^ CD3^-^ B220^-^ cells and total monocytes defined as CD11b^+^ Ly6G^-^ MERTK^-^ MHC IA/IE^-^ CD3^-^ B220^-^ cells. (**E**) Frequency of Ly6G^+^ CD11b^+^ CD3^-^ B220^-^ neutrophils was measured in BAL fluid of hFcγR mice that were pre-treated with chemokine neutralizing mAbs (anti-CXCL1 and anti-CCL3) or isotype control followed by administration of afucosylated immune complexes. The median and the 95% confidence interval are shown in each graph. P values in (A to D) were calculated using a one-way ANOVA with Dunnett’s correction using n=3 mice per group for A and B and n=4 mice per group for (C to E). Data in (A to E) are representative of at least two independent experiments. *P < 0.05; **P < 0.01; ***P < 0.001; ns, not significant.

BAL fluid collected from the lungs of mice that were treated with afucosylated ICs (pool 1) had elevated frequencies of neutrophils and monocytes over BAL fluid from mice treated with fucosylated ICs (pool 2) or mRNA-vaccine elicited ICs (pool 3) (Fig. 4A, fig. S5). BAL fluid from mice that received afucosylated ICs was also distinguished from all other experimental conditions by increased concentrations of proinflammatory cytokines and chemokines. Tumor necrosis factor (TNF)-α, interleukin (IL)-6, chemokine C-C motif ligand (CCL)-3, CCL4, chemokine C-X-C motif ligand (CXCL)-1, and CXCL10 were uniquely up-regulated whereas no difference was observed in concentrations of the immunoregulatory or immunosuppressive cytokine IL-10 (Fig. 4B). Collectively, these findings functionally distinguish afucosylated IgG, characteristic of severe COVID-19, from the highly sialylated and fucosylated, vaccine-elicited antibody glycoforms in vivo.

We next assessed the FcγR dependence of the immune response to afucosylated ICs. Mice specifically lacking expression of CD16a (CD16a^−/−^), but expressing all other human FcγRs, did not exhibit a similar inflammatory response to afucosylated ICs as mice expressing the complete human repertoire (WT) (Fig. 4C and D). This showed that the inflammatory potential of afucosylated IgG1 was highly dependent on the presence of CD16a-expressing immune effector cells. Because CXCL1 and CCL3 are known neutrophil chemoattractants, we next asked whether these molecules mediated the neutrophil influx after afucosylated IC administration. Indeed, pre-administration of blocking monoclonal antibodies against the chemokines CXCL1 or CCL3 led to a reduction in neutrophil recruitment (Fig. 4E) (*30*, *31*). Together, these findings support a mechanism in which afucosylated ICs in the lung trigger CD16a-dependent production of chemokines which promote subsequent influx of innate immune cells.

## DISCUSSION

Prognostic biomarkers and treatments that may halt the progression to severe COVID-19 are urgently needed to prevent mortality associated with this disease. To identify new avenues of treatment, mechanisms underlying the distinct trajectories in COVID-19 must be clarified. Here, we show that early antibody quality and the expression of cognate FcγRs on peripheral monocytes may be used to anticipate distinct COVID-19 trajectories, including progression to more severe outcomes. Overall, mild COVID-19 patients who experienced a worsening disease trajectory were characterized by the absence of an early robust neutralizing antibody response with elevations in both afucosylated anti-spike IgG and the CD16a receptor on myeloid cells. The IgG elicited by SARS-CoV-2 infection was heterogeneous in Fc glycosylation relative to IgG generated in response to SARS-CoV-2 mRNA vaccination. Vaccine-elicited IgG exhibited high neutralization and low afucosylation, along with other substantial differences in Fc glycoforms. The early inflammatory response to ICs in the lung was a function of the abundance of IgG1 afucosylation and CD16 expression.

Our data support a model in which the combination of a lack of early SARS-CoV-2 neutralization and an enhanced afucosylated IgG-CD16a signaling axis contribute to the inflammatory phenotype of severe COVID-19. We propose that this may be one mechanism contributing to the hyperinflammatory response in severe COVID-19. Determining how various immune aberrancies, including those described here and others such as elevated IL-6 or an impaired renin-angiotensin system might contribute to the pathogenesis of severe COVID-19 will require the development of new animal models (*2*, *24*, *32*, *33*). To examine whether afucosylated ICs can augment the inflammatory milieu in the lungs, we established a model to specifically evaluate the impact of human IgG signaling on the pulmonary inflammatory response. This model advances our ability to evaluate human IgG antibodies in a functional dimension, beyond what in vitro approaches can reveal. We show that the afucosylated IgG-CD16a signaling axis can result in a remodeling of the inflammatory lung milieu. Of note, the increased frequency of neutrophils and monocytes observed within the lungs of mice that received afucosylated ICs mirrors what has been observed in some severe COVID-19 patients (*4*, *21*, *34*, *35*). Tissue-resident alveolar macrophages likely serve as an initial effector of afucosylated IC activity in this model as they are the predominant innate immune cell population within the lung, exhibit high expression of CD16a, and can produce many of the observed soluble factors (*11*). This in vivo model is not a model of COVID-19 pathogenesis; rather, it enables a more targeted investigation of how distinct human antibody repertoires activate effector cells and the complex molecular changes involved in those interactions specifically within the lung. Animal models that more accurately reflect the immunophenotype of patients at highest risk for mortality in COVID-19 are needed to truly study the pathogenesis of this disease.

Although we did not observe a correlation between IgG afucosylation and the demographic features studied here, it is known that IgG post-translational modifications are associated with specific patient characteristics, including sex and age (*36*). Thus, differences in demographics between our cohorts may have contributed to our findings. How IgG glycosylation is regulated is not fully understood, but numerous studies support a role for both heritable and non-heritable influences (*29*, *37*–*41*). Our data support a direct role for plasmablast FUT8 expression as a determinant of IgG afucosylation. Defining specific regulatory pathways of IgG glycosylation will be important for modulating the in vivo activities of IgG to improve human disease outcomes.

This study has limitations. First, some patient demographic features differed between cohorts, particularly the distribution of sex and age in hospitalized patients as compared to outpatients and healthy vaccine recipients. Although statistical analysis performed here does not support sex and age as strong contributors to afucosylated IgG1 abundance, we cannot definitively conclude a lack of contribution from these features or other undetermined variables. Second, both independent COVID-19 outpatient cohorts included only a small number of progressors, leading to unequal sample sizes. Our identification of an association between increased afucosylated IgG1 and COVID-19 progression may draw greater attention to and assessment for this antibody modification in additional patient cohorts which may assist in the validation of increased afucosylated IgG1 as a potential prognostic marker of progressive COVID-19. That said, and as a third limitation, there are currently no clinical assays to assess IgG1 afucosylation. The development of one such high-throughput, clinical assay could dramatically increase assessment for afucosylated IgG1 in a variety of diseases and increase consistency in method of assessment between groups (*42*, *43*). Fourth and finally, the in vivo model described here is not a model of severe COVID-19 pathogenesis. The development of animal models that more faithfully recapitulate the risk factors and immune responses associated with severe disease in humans are necessary in this endeavor.

In conclusion, in this study of two independent COVID-19 outpatient cohorts, an early, non-neutralizing, afucosylated antibody response was observed to be associated with COVID-19 symptom progression. These findings begin to suggest that an early assessment for non-neutralizing, afucosylated IgG1 may be able to identify those patients at risk of developing severe disease in response to SARS-CoV-2 infection or infection by other viruses.

## MATERIALS AND METHODS

### Study Design

The overall objectives of this study were to characterize the pre-progressive antibody responses during early, mild COVID-19 and to identify antibody characteristics associated with distinct disease outcomes and SARS-CoV-2 mRNA vaccination. To this end, we studied samples from two independent cohorts of mild COVID-19 patient enrolled in phase 2 clinical trials. We evaluated data only from the placebo arm of both studies so that our findings are not impacted by the experimental treatments trialed in either study. All subjects were assessed for pseudoviral neutralization at least in duplicates, and IgG glycosylation were quantified for all patients who progressed to severe disease (progressors), hospitalized patients and from a subset of randomly selected non-progressors and mRNA vaccinees a (investigators were blinded). As progressors were identified post hoc, study size calculations were not performed. No selection criteria were used to select the hospitalized patients from Mount Sinai other than their status as hospitalized COVID-19 patients. The mRNA vaccine recipient cohort participants were selected based on having no known prior exposure to SARS-CoV-2 and a recent negative PCR test result for SARS-CoV-2. No other selection criteria were used and there were no outliers. Investigators were not blinded to patient status.

For the animal studies, no statistical methods were employed to predetermine sample size. Mice were randomized to achieve equal distribution of age (8 to 12 weeks) and sex (1 to 1, male to female). Treatment groups were consistently blinded to the person involved in treatment administration and tissue processing. Cellular, cytokine, and chemokine measurements were performed in duplicate and data are representative of 2-3 independent experiments.

### Clinical cohorts and samples

Characterization of these samples at Stanford was performed under a protocol approved by the Institutional Review Board of Stanford University (protocol #55718). For the Stanford Lambda cohort (Cohort 1), 120 participants were enrolled in a phase 2 randomized controlled trial of Peginterferon Lambda-1a beginning April 25, 2020 (Lambda, NCT04331899). Inclusion/exclusion criteria and the study protocol for the trial have been published (*15*). Briefly, adults aged 18 to 75 years old were enrolled within 72 hours of testing positive for SARS-CoV-2 by an FDA emergency use authorized RT-PCR within 72 hours prior to enrollment were eligible for study participation. Exclusion criteria included hospitalization, respiratory rate >20 breaths per minute, room air oxygen saturation <94%, pregnancy or breastfeeding, decompensated liver disease, recent use of investigational or immunomodulatory agents for treatment of COVID-19, and prespecified lab abnormalities. All participants gave written informed consent, and all study procedures were approved by the Institutional Review Board of Stanford University (IRB-55619). Participants were randomized to receive a single subcutaneous injection of Lambda or saline placebo. Peripheral blood was collected at enrollment, day 5, and day 28 post enrollment. A subset of participants (n=80) returned for long-term follow-up visits 4-, 7-, and 10-months post enrollment, with peripheral blood obtained. Longitudinal samples from the 56 SARS-CoV-2-infected outpatients who were in the placebo arm of the broader Lambda study were obtained and assessed here.

For the Stanford Favipiravir Cohort (Cohort 2), 149 participants were enrolled in a phase 2 randomized controlled trial of Favipiravir beginning July 12, 2020 (NCT04346628). Inclusion/exclusion criteria and the study protocol for the trial are publicly available. Briefly, adults aged 18 to 80 years old were enrolled within 72 hours of a positive nucleic acid amplification test for SARS-CoV-2. Upon enrollment, participants were mildly symptomatic with no evidence of respiratory distress. Participants were randomized to receive favipiravir or placebo. Participants were followed for 28 days, with study visits on days 1, 5, 10, 14, 21 and 28. At each study visit, clinical assessment was performed and oropharyngeal swabs and blood samples were collected. Samples collected upon enrollment from the 69 SARS-CoV-2-infected outpatients who were in the placebo arm of the broader Favipiravir study were obtained and assessed here.

For the cohort of patients hospitalized with COVID-19, 52 samples were obtained from hospitalized COVID-19 patients enrolled in the Mount Sinai Health System (MSHS) collected by the Mount Sinai COVID-19 biobank (*2*). The median age was 65 years old with a range from 33 to 98 years old. There were 31 males and 21 females in the study. 13 patients succumbed to disease.

For the Stanford adult vaccine cohort, 57 healthy volunteers were enrolled in the study approved by Stanford University Institutional Review Board (IRB 8629). The median age was 36 years old with a range from 19 to 79 years old. There were 28 males and 29 females in the study. There were 27 White participants, 23 Asian participants, 4 Black participants, 1 Native American participant, and 2 other participants.

### Cell lines

Human embryonic kidney (HEK) 293T (American Type Culture Collection, ATCC; CRL-3216) and Vero (ATCC; CCL-81) cells were used in this study. Cells were grown and maintained in 1X Dulbecco’s Modified Eagle Medium (DMEM; Thermo Fisher Scientific). Media was supplemented with 10% fetal bovine serum (FBS).

### Cloning, expression, and protein purification

The His_6_-tagged SARS-CoV-2 receptor binding domain (RBD) and full-length SARS-CoV-2 spike protein were purified in house as previously described (*9*). Both constructs were transiently transfected into Expi293F cells (Thermo Fisher Scientific). Proteins were purified from culture supernatants using Ni-nitriloacetic acid (NTA) resin (GE HealthCare).

### Generation of SARS-CoV-2 pseudoparticles

To generate vesicular stomatitis virus (VSV) pseudotyped with the spike protein of SARS-CoV-2, we first constructed an expression plasmid encoding the WT SARS-CoV-2 spike protein. We did this by modifying a pCAGGS mammalian expression vector encoding the full-length WT spike protein and deleting its last 18 amino acids of the cytoplasmic domain, which we call pCAGGS-S∆18. This reagent was produced under HHSN272201400008C and obtained through Biodefense and Emerging Infections (BEI) Resources, National Institute of Allergy and Infectious Disease (NIAID), National Institutes of Health (NIH): Vector pCAGGS containing the SARS-related coronavirus 2, Wuhan S, NR52310. To generate VSV pseudotyped with SARS-CoV-2 spike protein, we first coated 6-well plates with 0.5 μg/mL poly-D-lysine (Thermo Fisher Scientific, Cat. No. A3890401) for 1 to 2 hours at room temperature. After poly-D-lysine treatment, plates were washed three times with sterile water and then seeded with 1.5x10^6^ HEK 293T cells per well. After 24 hours, cells were transfected with 1 μg of pCAGGS-S∆18 per well using Lipofectamine 2000 transfection reagent (Thermo Fisher Scientific, Cat. No., 11668019). Forty-eight hours after transfection, the cells were washed once with 1X phosphate buffered saline (PBS) and were infected with VSV-∆G-green fluorescent protein (GFP)/nanoluciferase (a generous gift from Matthias J. Schnell) at a multiplicity of infection of 2 to 3 in a 300 μL volume. Cells were infected for an hour with intermittent rocking every 15 min. After infection, the inoculum was carefully removed, and the cell monolayer was washed three times with 1X PBS to remove residual VSV-∆G-GFP/nanoluciferase. Two mL of infection media (2% FBS, 1% glutamine, 1% sodium pyruvate in 1X DMEM) was added to each well. At 24 hours post-infection, the supernatants from all the wells were combined, centrifuged (600 *g* for 10 min, 4°C), and stored at -80°C until use.

### Neutralization assays

Vero cells were seeded at 5x10^5^ cells per well in 50 μL aliquots in half area Greiner 96-well plates (Greiner Bio-One; Cat. No. 675090) 24 hours prior to performing the neutralization assay. On separate U-bottom plates, patient plasma samples were plated in duplicates and serially 5-fold diluted in infection media (2% FBS, 1% glutamine, 1% sodium pyruvate in 1X DMEM) for a final volume of 28 μL per well. We also included ‘virus only’ and ‘media only’ controls. Twenty-five microliters of SARS-CoV-2 pseudo-typed VSV particles (containing 500 to 1500 fluorescent forming units) were added to the wells on the dilution plate, not including the “virus-free” column of wells and incubated at 37°C for 1 hour. Prior to infection, Vero cells were washed twice with 1X PBS and then 50 μL of the incubated pseudo-typed particles, and patient plasma mixture was then transferred from the U-bottom 96-well dilution plates onto the Vero cells and placed into an incubator at 37°C and 5% CO2. At 24 hours post-incubation, the number of GFP-expressing cells indicating viral infection were quantified using a Celigo Image Cytometer. We first calculated percent infection based on our ‘virus only’ controls and then calculated percent inhibition by subtracting the percent infection from 100. A non-linear curve and the half-maximal neutralization titer (pNT50) were generated using GraphPad Prism.

### Enzyme-linked immunosorbent assay (ELISA)

ELISAs were performed following a modified version of a protocol described previously (*9*). Briefly, 96 Well Half-Area microplates (Corning (Millipore Sigma)) were coated with antigens at 2μg/ml in PBS for 1 hour at room temperature. Next, the plates were blocked for an hour with 3% non-fat milk in PBS with 0.1% Tween 20 (PBST). All serum samples from patients with COVID-19, and the negative controls, were heated at 56°C for 1 hour, aliquoted and stored at -80°C. Serum samples were diluted 5-fold starting at 1:50 in 1% non-fat milk in PBST. Diluted serum samples (25μl) were added to each well and incubated for 2 hours at room temperature. Following primary incubation with the serum, 25μl of 1:5000 diluted horseradish peroxidase (HRP)-conjugated anti-Human IgG secondary antibody (Southern Biotech, cat# 2040-05) was added and incubated for 1 hour at room temperature. The plates were developed by adding 25μl per well of the chromogenic substrate 3,3′,5,5′-tetramethylbenzidine (TMB) solution (Millipore Sigma). The reaction was stopped with 0.2N sulphuric acid (Sigma-Aldrich) and absorbance was measured at 450nm (SPECTRAmax iD3, Molecular Devices). The plates were washed 5 times with PBST between each step and an additional wash with PBS was done before developing the plates. All data were normalized between the same positive and negative controls and the binding area under the curve (AUC) has been reported.

### IgG Fc glycan analysis.

Methods for relative quantification of Fc glycans and IgG subclasses have been previously described (*9*, *29*). Briefly, IgG were isolated from serum by protein G purification. Antigen-specific IgG were isolated on NHS agarose resin (Thermo Fisher Scientific; 26196) coupled to the protein of interest. Following tryptic digestion of purified IgG bound to antigen-coated beads, nanoscale liquid chromatography coupled to tandem mass spectrometry (nano LC-MS/MS) analysis for characterization of glycosylation sites was performed on an UltiMate3000 nanoLC (Dionex) coupled with a hybrid triple quadrupole linear ion trap mass spectrometer, the 4000 Q Trap (SCIEX). MS data acquisition was performed using Analyst 1.6.1 software (SCIEX) for precursor ion scan triggered information dependent acquisition (IDA) analysis for initial discovery-based identification.

For quantitative analysis of the glycoforms at the N297 site of IgG1, multiple-reaction monitoring (MRM) analysis for selected target glycopeptides and their glycoforms was applied using the nanoLC-4000 Q Trap platform to the samples which had been digested with trypsin. The m/z of 4-charged ions for all different glycoforms as Q1 and the fragment ion at m/z 366.1 as Q3 for each of transition pairs were used for MRM assays. A native IgG tryptic peptide (131-GTLVTVSSASTK-142) with Q1/Q3 transition pair of, 575.9^+2^/780.4 was used as a reference peptide for normalization. IgG subclass distribution was quantitatively determined by nanoLC-MRM analysis of tryptic peptides following removal of glycans from purified IgG with PNGase F. Here the m/z value of fragment ions for monitoring transition pairs was always larger than that of their precursor ions with multiple charges to enhance the selectivity for unmodified targeted peptides and the reference peptide. All raw MRM data was processed using MultiQuant 2.1.1 (SCIEX). All MRM peak areas were automatically integrated and inspected manually. In the case where the automatic peak integration by MultiQuant failed, manual integration was performed using the MultiQuant software.

### Immune cell phenotyping and FcγR quantification

Cryopreserved human PBMCs collected upon enrollment on study day 0 were rapidly thawed, washed, and blocked with Human TruStain FcX (BioLegend; cat# 422302) to reduce nonspecific binding. Cells were then stained for viability with Live/Dead Fixable Aqua Staining Kit (Thermo Fisher Scientific; cat# L34957) as well as with combinations of the following antibodies for 20 min at 4°C: Alexa Fluor (AF) 700 anti-CD3 (clone OKT3; cat# 317340), allophycocyanin (APC)/Fire750 anti-CD11c (clone S-HCL-3; cat# 371510), Brilliant Violet (BV) 785 anti-CD14 (clone M5E2; cat# 301840), BV421 anti-CD16 (clone 3G8; cat# 302038), AF700 anti-CD19 (clone SJ25C1; cat# 363034), anti-phycoerythrin (PE) CD21 (Bu32; cat# 354904), BV785 anti-CD27 (clone O323; cat# 302832), fluorescein isothiocyanate (FITC) anti-CD32 (STEMCELL Technologies; clone IV.3; cat# 60012FI), APC anti-CD32B/C (clone S18005H; cat# 398304), FITC anti-CD38 (clone S17015A; cat# 397108), PE anti-CD56 (clone 5.1H11; cat# 362508), peridinin-chlorophyll-protein (PerCP)-Cy5.5 anti-CD138 (clone MI15; cat# 356510), APC-Cy7 anti-IgD (clone IA6-2; cat# 348218), AF647 anti-FucT-VIII (Santa Cruz Biotechnologies; clone B-10; cat# sc-271244 AF647), and BV650 anti-HLA-DR (clone L243; cat# 307650) purchased from BioLegend unless noted otherwise. After staining, cells were washed, resuspended in fixation buffer (BioLegend; cat# 420801), and acquired using an Attune NxT flow cytometer (Invitrogen). In the case of intracellular anti-FucT-VIII staining, cells were further permeabilized using Intracellular Staining Permeabilization Wash Buffer (BioLegend; cat# 421002) prior to acquisition by flow cytometry. Bulk myeloid cells were defined as viable CD3^-^ CD19^-^ CD56^-^ CD11c^+^ HLA-DR^+^ cells, and CD16a^+^ monocytes within this population were additionally positive for CD16a (fig. S3). Within CD16a^+^ monocytes, non-classical (NC) monocytes were CD16a^+^ CD14^-^, and intermediate (int) monocytes were CD16a^+^ CD14^+^. Leukocyte expression of FcγRs was quantified by measuring the median fluorescence intensity (MFI) of a particular FcγR and comparing it to the MFI of stained Quantum Simply Cellular microsphere beads (Bangs Laboratories) of known and discrete antibody-binding capacities. Total CD19^+^ B cells were similarly assessed from within viable PBMCs. Plasmablasts were further defined as CD19^+^ CD27^+^ CD38^++^. Memory B cells were defined as CD19^+^ CD27^+^ IgD^-^, double negative (DN) B cells were CD19^+^ CD27^-^ IgD^-^, and naïve B cells were CD19^+^ CD27^-^ IgD^+^.

### In vivo lung inflammation model

All in vivo experiments were performed in compliance with federal laws and institutional guidelines and have been approved by the Stanford University Institutional Animal Care and Use Committee. Polyclonal IgG was isolated from plasma from patients who were PCR-positive for SARS-CoV-2, pooled based on the frequency of afucosylated anti-RBD IgG1 (>20% or <10%). Similarly, plasma from all vaccinated patient samples were pooled and IgG was purified. The purified IgG pools were incubated with SARS-CoV-2 spike trimer at a 20:1 molar ratio overnight at 4°C. Immune complexes were intratracheally administered to 8 to 12-week-old, sex-matched, fully FcγR humanized or CD16a-deficient C57BL/6 mice (*14*). Experimental groups were consistently matched for sex and age, but otherwise randomized. Four hours post-administration, mice were euthanized and bronchoalveolar lavage (BAL) was performed. Immune cells were isolated from within the BAL fluid, blocked with Human TruStain FcX (BioLegend; cat# 422302) to reduce nonspecific binding, and stained with the following cell staining panel for 20 min at 4°C: Live/Dead Aqua Fixable Dye (Thermo Fisher Scientific; cat# L34957), PerCP-Cy5.5 anti-CD3 (clone 17A2; cat# 100218), BV650 anti-CD11b (clone M1/70; cat# 101259), AF700 anti-CD45 (clone I3/2.3; cat# 147716), PerCP-Cy5.5 anti-B220 (clone RA3-682; cat# 103236), APC anti-Ly6C (clone HK1.4; cat# 128016), BV785 anti-Ly6G (clone 1A8; cat# 127645), PE anti-MERTK (clone 2B10C42; cat# 151506), and APC/Fire 750 anti-MHC II (clone M5/114.15.2; cat# 107652) purchased from BioLegend unless otherwise noted. Once stained, cells were washed, resuspended in fixation buffer (BioLegend; cat# 420801), and acquired using an Attune NxT flow cytometer (Invitrogen). Neutrophils were defined as viable Ly6G^+^ CD11b^+^ CD3^-^ B220^-^ leukocytes. Monocytes were defined as viable CD11b^+^ Ly6G^-^ MERTK^-^ MHC IA/IE^-^ CD3^-^ B220^-^ leukocytes (fig. S5). Cell-free BAL fluid was stored at 4°C and processed within 24 hours to measure cytokine and chemokine content using a LEGENDplex bead array kits (BioLegend; cat# 740390 and 740451). In chemokine-blockade experiments, mice received intraperitoneal injections of 5mg/kg anti-CXCL1, anti-CCL3, or rat IgG2a isotype control (R&D Systems; clones 48415, 756605, 54447; cat# MAB453, MAB4502, MAB006) 8 hours prior to immune complex administration and immune complex administration and BAL were performed as described above. Researchers were blinded to experimental groups and agents throughout these in vivo studies.

### Statistical Analysis

The log10+1 transformed half-maximal serum neutralization titers (pNT50) were used to generate the heatmap. Python version 3.8.5 was used for machine learning using open-source scikit-learn package (*44*). The class progressor was mapped to 1 and non-progressor was mapped to 0, making it a binary classification problem. To determine whether the combination of low/no neutralizing antibodies and elevated IgG Fc afucosylation was a predictor of worsening disease trajectory, a SVM classifier was used. The model was trained using data from Cohort 1 (training set), and to obtain the best hyperparameters, GridSearch cross-validation (cv) was performed. The model was tested using an independent test set (Cohort 2) and the ROC AUC score was generated. To generate ROC AUC scores from FcγRs frequency and expression to distinguish progressors and non-progressors, Random Forest Classifier was used. The input data was split using 6-fold cross validation in which the classifier was trained on 5 folds of the data and tested on the remaining part of the data. The ROC response for all these different datasets were used for calculating the mean area under curve.

R Studio (version 1.2.1335) was used to perform the multivariate regression analyses and to generate the radar plots and bubble plot using ggplot2 package. For the radar plots, each feature was normalized across the entire dataset and the mean value within each cohort (progressor and non-progressor) was plotted. For the bubble plot, cytokine and chemokine concentrations were normalized between 0 and the average of all values across all the groups. All other data were analyzed with GraphPad Prism 9.0 software. For pairwise comparisons, unpaired *t* tests with Welch’s correction or Wilcoxon rank-sum test was used. For multiple comparisons between unrelated groups, one way analysis of variance (ANOVA) with Tukey’s correction, one-way ANOVA with Dunnett’s correction, two-way (ANOVA) with Tukey’s correction, and Brown-Forsythe and Welch ANOVA test with Dunnett T3 correction was used based on the data. For multiple comparisons between matched data mixed effect analysis with Geisser-Greenhouse and Tukey’s correction was implemented.

## Supplementary Materials

### Other Supplementary Material for this manuscript includes the following:

Data file S1

MDAR Reproducibility Checklist
