# Supplementary material for: Early non-neutralizing, afucosylated antibody responses are associated with COVID-19 severity
Source: Sci Transl Med. 2022 Jan 18:eabm7853. doi: 10.1126/scitranslmed.abm7853 (PMC8939764; doi:10.1126/scitranslmed.abm7853)
Supplement: Supplementary file 1 — Figs. S1 to S5 Tables S1 to S4 [file scitranslmed.abm7853_sm.pdf]

Science Translational Medicine
AAAS

Supplementary Materials for

Early non-neutralizing, afucosylated antibody responses are associated with COVID-19 severity

Saborni Chakraborty *et al.*

Corresponding author: Taia T. Wang, taiawang@stanford.edu

DOI: 10.1126/scitranslmed.abm7853

The PDF file includes:

Figs. S1 to S5
Tables S1 to S4

Other Supplementary Material for this manuscript includes the following:

Data file S1
MDAR Reproducibility Checklist

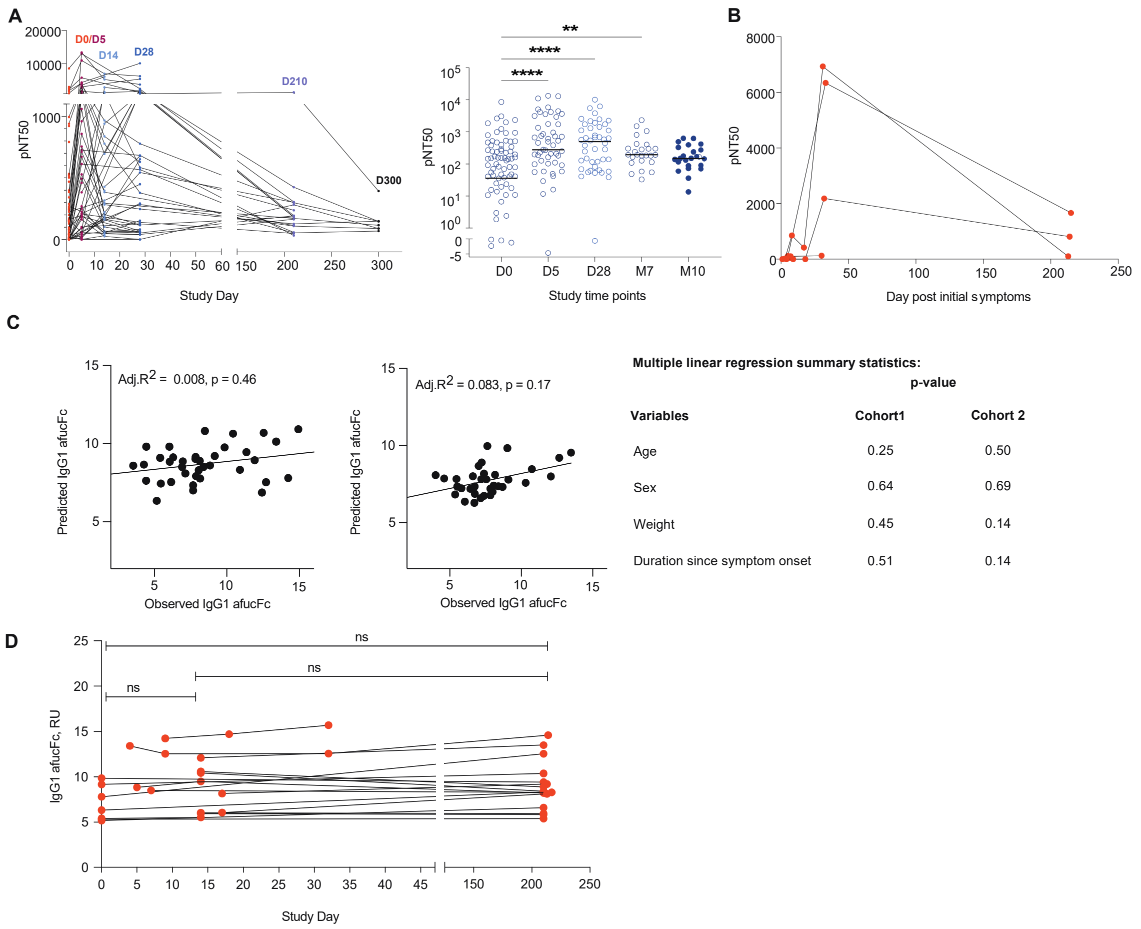

Multiple linear regression summary statistics:

Variables	p-value Cohort1	p-value Cohort 2
Age	0.25	0.50
Sex	0.64	0.69
Weight	0.45	0.14
Duration since symptom onset	0.51	0.14

Fig. S1. Neutralizing titers and abundance of IgG1 Fc afucosylation in COVID-19 outpatients over time. (A) Half-maximal SARS-CoV-2 pseudovirus neutralizing titers (pNT50) are shown for each study time point. Left panel: graphed to match format of Figure 1A. Right panel: plotted to clearly show the median pNT50 at each study time point. Horizontal bars indicate median. **(B)** The kinetics of neutralizing antibody response are shown over time in progressors in Cohort 1. **(C)** Multivariate regression analysis was used to test the contribution of age, sex, weight, or duration since COVID-19 onset on abundance of afucosylated anti-RBD IgG1 in two cohorts **(D)** Abundance of SARS-CoV-2 specific afucosylated IgG1 (afucFc) is shown for Cohort 1 COVID-19 outpatients over time (n=18). RU, relative units. P values in (A) and (D) were calculated using Kruskal Wallis test with Dunn's correction for multiple comparisons. **P < 0.01; ****P < 0.0001; ns, not significant.

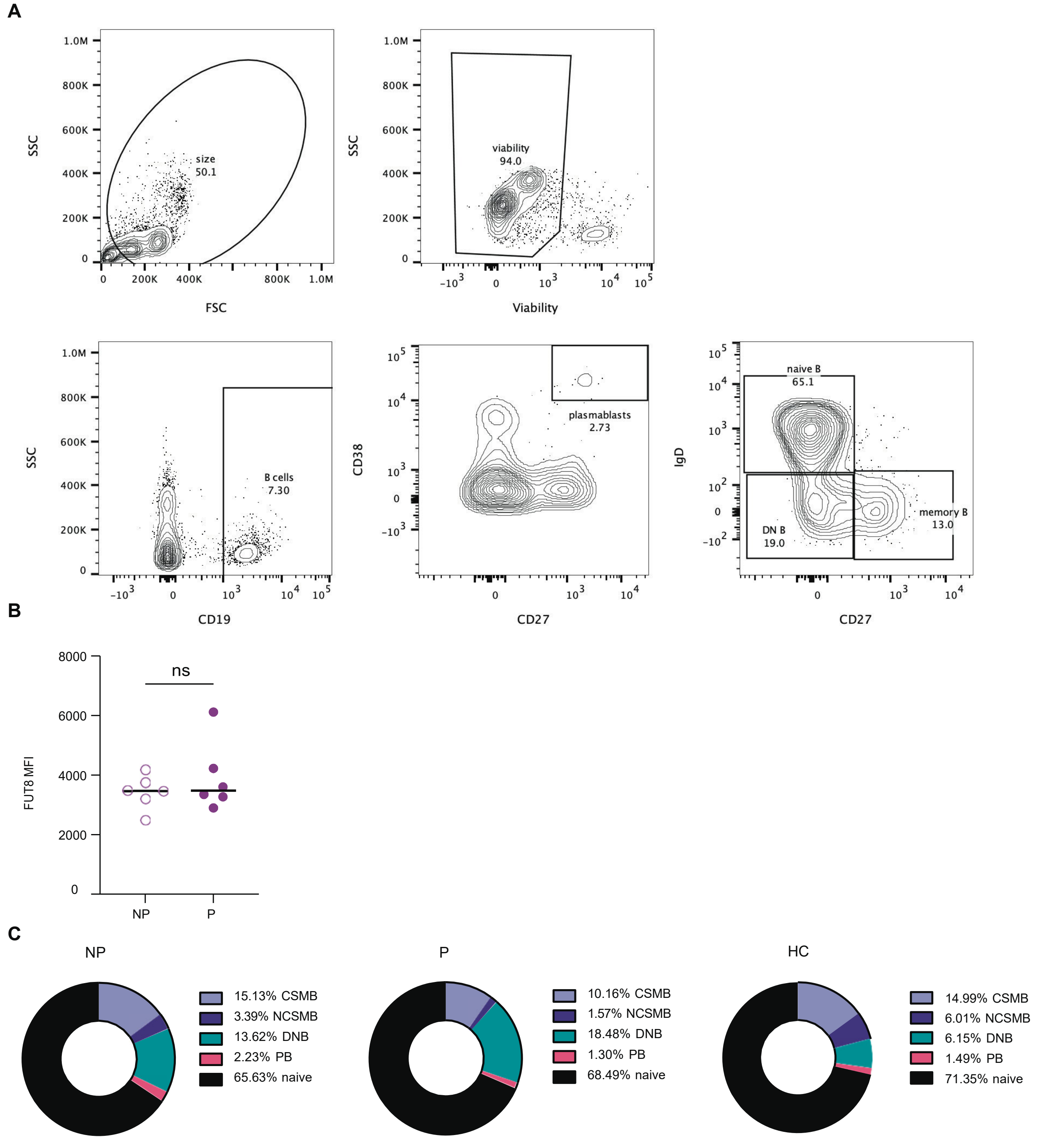

Fig. S2. Characterization of B cell subsets in COVID-19 outpatients. (A) The gating strategy for B cell subsets is shown. Total CD19^{+} B cells were assessed from within viable peripheral blood mononuclear cells (PBMCs). Plasmablasts were further defined as CD19^{+} CD27^{+} CD38^{++}. Memory B cells were defined as CD19^{+} CD27^{+} IgD^{-}, double negative (DN) B cells were CD19^{+} CD27^{-} IgD^{-}, and naïve B cells were CD19^{+} CD27^{-} IgD^{+}. FSC, forward scatter; SSC, side scatter. **(B)** FUT8 expression is shown within total viable PBMCs in progressors (P; n =6) and sex-matched non-progressors (NP; n=6). MFI, median fluorescence intensity. Horizontal bars indicate median. **(C)** Distribution of B cell subsets (class switched memory B cells (CSMB), non-class switched memory B cells (NCSMB), CD27^{-} IgD^{-} double negative B cells (DNB), plasmablasts (PB), and naïve B cells in progressors (P; n = 6), non-progressors (NP, n = 6), and healthy controls (HC, n = 4) are shown. P values in (B) was calculated using unpaired Student's t test; ns, not significant.

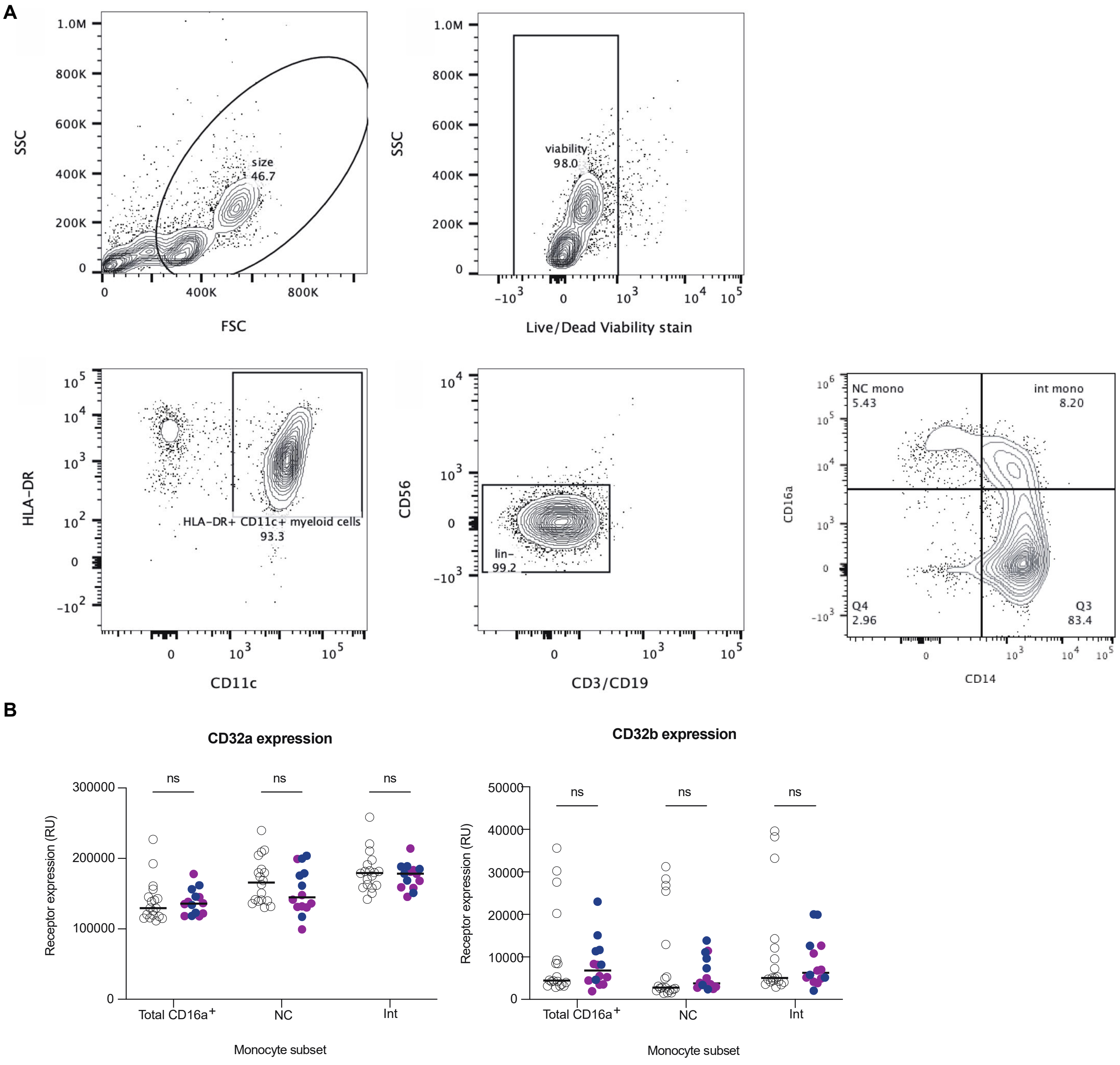

Fig. S3. Characterization of monocyte subsets in COVID-19 outpatients. (A) The gating strategy for day 0 monocyte subsets and FcγR expression is shown. Bulk myeloid cells were defined as viable $CD3^{-}$ $CD19^{-}$ $CD56^{-}$ $CD11c^{+}$ HLA-DR^{+} cells, while $CD16a^{+}$ monocytes within this population were additionally positive for CD16a. Within $CD16a^{+}$ monocytes, non-classical (NC) monocytes were $CD16a^{+}$ $CD14^{-}$ while intermediate (Int) monocytes were $CD16a^{+}$ $CD14^{+}$. **(B)** FcγR (CD32a and CD32b) expression on myeloid cell subsets is shown. Horizontal bars indicate median. P values were determined by unpaired Student's test. ns, not significant.

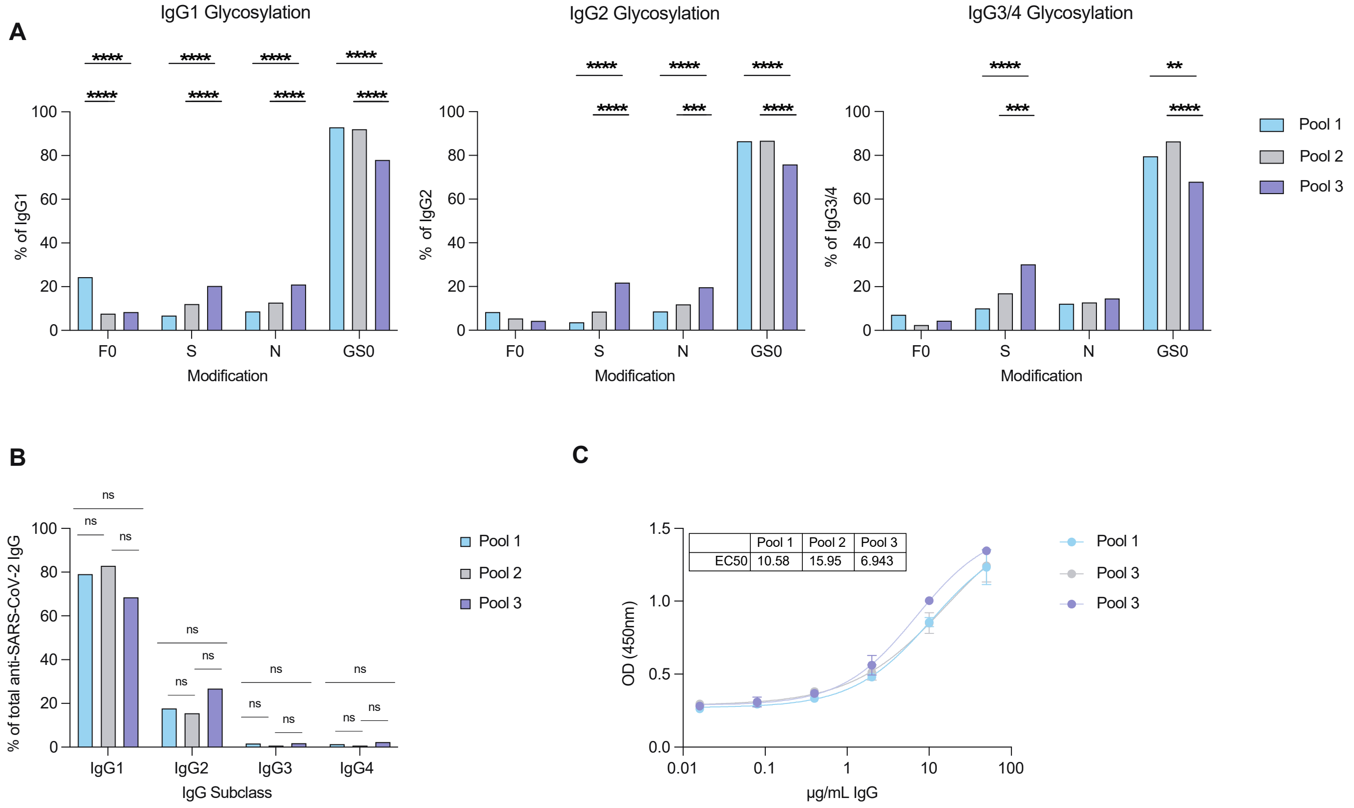

Fig. S4. Characterization of polyclonal IgG pools. (A) Glycan composition (F0-afucosylation, GS0-Galactosylation, S-Sialylation and N-Bisection) of IgG1, IgG2 and IgG3/4 was measured for individually purified patient IgG comprising polyclonal pool 1 (F0, F0>20%), pool 2 (F, F0<10%), and pool 3 (Vax). **(B)** IgG subclass distribution is shown for the individually purified patient IgG comprising polyclonal pool 1, pool 2, and pool 3. **(C)** SARS-CoV-2 full length spike binding to polyclonal pool 1, pool 2, and pool 3 is shown; OD, optical density. Data in (C) are presented as mean ± SD; EC50, half maximal binding concentration. P values in (A and B) were calculated using two-way ANOVA with Sidak's correction. **P < 0.01; ***P < 0.001; ****P < 0.0001; ns, not significant.

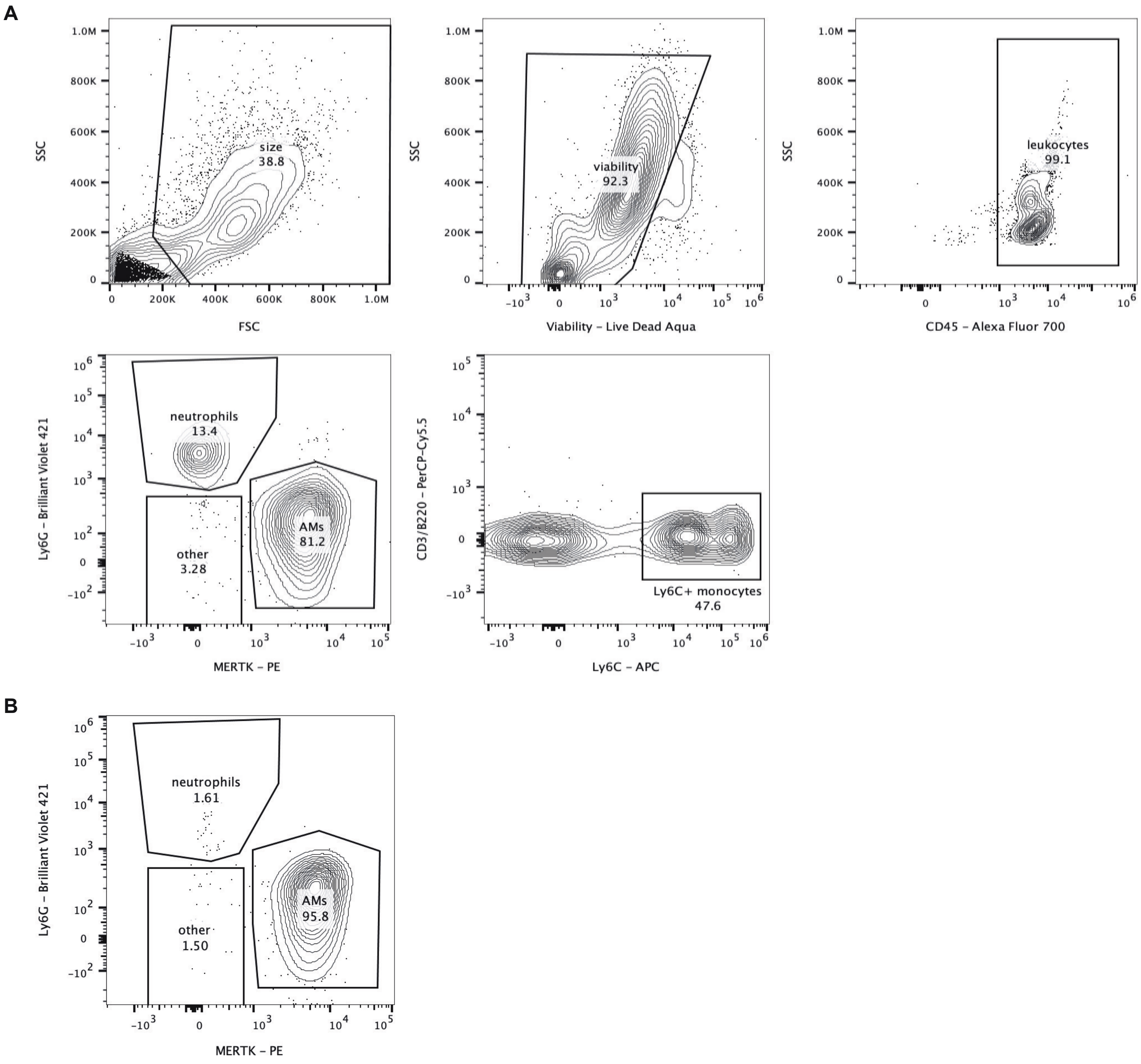

Fig. S5. Gating strategy for immune cell infiltrates in mouse BAL. (A) Neutrophils were definedas viable Ly6G$^+$ CD11b$^+$ CD3$^-$ B220$^-$ leukocytes. Monocytes were defined as viable CD11b$^+$ Ly6G$^-$ MERTK$^-$ MHC IA/IE$^-$ CD3$^-$ B220$^-$ or Ly6C$^+$ CD11b$^+$ Ly6G$^-$ MERTK$^-$ MHC IA/IE$^-$ CD3$^-$ B220$^-$ leukocytes. **(B)** Alveolar macrophages were magnetically sorted based on positive expression of MERTK. This sorting method consistently resulted in >90% purity, as determined by flow cytometry. PE, phycoerythrin; APC, allophycocyanin.

Tables

Table S1. Demographics of the COVID-19 infection cohorts

**Stanford Lambda cohort (Cohort 1)**	**Non-progressor (n=102)**	**Missing values**	**Progressor (n=8)**	**Missing values**	**p value**
Age, years (median, 95%CI)	36 (32.8-38.2)	0	45 (34.4-55.7)	0	ns
Sex Female	45 (44.1%)	0	3 (38%)	0	ns
Race/Ethnicity		0		0	
Latinx	65 (63.7%)		5 (62.5%)		
White	29 (28.4%)		2 (25%)		
Asian	7 (6.9%)		0 (0%)		
Others	1(1%)		1 (12.5%)		
Weight (lbs) (median, 95% CI)	175 (168.1-181.9)	1	198.5 (148.8-248.2)	0	ns
Body mass index (BMI) (median, 95% CI)	27.2 (26.5-28.9)	3	28.8 (23.9-33.7)	0	ns
<25	25 (25.3%)		2 (25%)		
25-30	38 (38.4%)		3 (37.5%)		
>30	36 (36.4%)		3 (37.5%)		
Median days from symptoms onset to study enrollment	4	17	3.5	0	ns
Mean days from symptoms onset to study enrollment	5	17	3.6	0	ns
Median days from randomization to disease resolution	9	0	16	4	ns

**Stanford Favipiravir Cohort (Cohort 2)**	**Non-progressor (n=67)**	**Missing values**	**Progressor (n=7)**	**Missing values**	**p value**
Age, years (median, 95%CI)	40 (36.9-43.1)	0	52 (45.9-58)	0	**
Sex Female	35 (52.2%)	0	3 (42.9%)	0	ns
Race/Ethnicity		4		0	
Latinx	26 (38.8%)		5 (71.4%)		
White	27 (40.2%)		1 (14.3%)		
Asian	7 (10.4%)		0 (0%)		
Others	3(4.5%)		1 (14.3%)		
Weight (lbs) (median, 95% CI)	180 (170.4-189.6)	0	190 (146.5-233.4)	0	ns
Body mass index (BMI) (median, 95% CI)	28.3 (26.8-29.7)	0	30.8 (24.7-37)	0	ns
<25	20 (29.9%)		2 (28.6%)		
25-30	21 (31.3%)		1 (14.3%)		
>30	26(38.8%)		4 (57.1%)		
Median days from symptoms onset to study enrollment	5	3	5	0	ns
Mean days from symptoms onset to study enrollment	5.7	3	6.4	0	ns
Median days from randomization to disease resolution	11	0	16	0	ns

**Mount Sinai Cohort**	**Hospitalized (n=52)**
Age, years (median, 95%CI)	65(60.6-69.4)
Sex Female	21(40.4%)
Race/Ethnicity	
Latinx	13 (25%)
White	7 (13.5%)
Asian	3 (5.8%)
Others	29 (55.8%)
Weight (lbs) (median, 95% CI)	N/A
Body mass index (BMI) (median, 95% CI)	26.79(24-6-29.01)
<25	15(28.8%)
25-30	21(40.4%)
>30	16(30.8%)
Median days of sample time point post hospitalization (95% CI)	4(2.87-5.13)

P values were calculated by comparing features between the progressors and non- progressors using unpaired t-tests with Welch's correction for categorical variables orFisher's t-test for continuous variables. CI, confidence intervals; lbs, pounds; N/A not available; ns, not significant

Table S2. Description of progressors from Cohort 1

Age	Sex	Days of symptoms prior to enrollment	Days to Hospitalization/ED visit post assessment of mild COVID-19
25	Female	3	0; progressive symptoms leading to hospitalization within 24-hours.
58	Male	4	5; progressively worsening respiratory symptoms. Referred to ED by study-associated physician on study day 5.
44	Female	3	1; progressively worsening respiratory symptoms.
34	Male	2	13; worsening symptoms.
47	Male	5	3; two ED visits within first 3 days for worsening respiratory symptoms.
59	Female	7	0; progressive symptoms leading to hospitalization within 24-hours.
28	Male	4	0; progressive symptoms leading to hospitalization within 24-hours.
46	Male	1	2; progressive symptoms leading to hospitalization.

Table S3. Description of progressors from Cohort 2

Age	Sex	Days of symptoms prior to enrollment	Days to Hospitalization/ED visit post assessment of mild COVID-19
44	Female	8	8; worsening respiratory symptoms
54	Male	2	1; sent by study team to ED for worsening respiratory symptoms and leg swelling, resulted in hospitalization
57	Male	4	2; sent by study team to ED for worsening respiratory symptoms; hospitalized
46	Male	5	3; worsening symptoms and new rash
63	Female	10	5; sent by study team to ED for worsening symptoms
52	Male	4	10; sent by study team to ED for worsening respiratory symptoms; hospitalized
50	Female	8	3; sent by study team to ED for worsening respiratory symptoms; hospitalized

Table S4: Demographics of the Stanford Adult Vaccination Cohort

**Characteristics**	
Age, years (median, 95%CI)	36(28.3-43.7)
Sex Female	11(64.7%)
Race/Ethnicity	
Latinx	0(0.0%)
White	7(41.2%)
Asian	8(47.1%)
Others	2(11.8%)
